# Lanthipeptides: chemical synthesis versus in vivo biosynthesis as tools for pharmaceutical production

**DOI:** 10.1186/s12934-016-0502-y

**Published:** 2016-06-07

**Authors:** Elvis Legala Ongey, Peter Neubauer

**Affiliations:** Chair of Bioprocess Engineering, Department of Biotechnology, Technische Universität Berlin, Ackerstraße 76, ACK24, 13355 Berlin, Germany

**Keywords:** Lanthipeptide, Bioprocess, Bioengineering, Industrial, Economic, Large-scale, Ruminococcin, Enfuvirtide

## Abstract

Lanthipeptides (also called lantibiotics for those with antibacterial activities) are ribosomally synthesized post-translationally modified peptides having thioether cross-linked amino acids, lanthionines, as a structural element. Lanthipeptides have conceivable potentials to be used as therapeutics, however, the lack of stable, high-yield, well-characterized processes for their sustainable production limit their availability for clinical studies and further pharmaceutical commercialization. Though many reviews have discussed the various techniques that are currently employed to produce lanthipeptides, a direct comparison between these methods to assess industrial applicability has not yet been described. In this review we provide a synoptic comparison of research efforts on total synthesis and in vivo biosynthesis aimed at fostering lanthipeptides production. We further examine current applications and propose measures to enhance product yields. Owing to their elaborate chemical structures, chemical synthesis of these biomolecules is economically less feasible for large-scale applications, and hence biological production seems to be the only realistic alternative.

## Background

The development of novel potent antimicrobial compounds to redress clinical problems caused by Staphylococci and Enterococci forms of bacteria remains a permanent challenge for humanity to combat the severe problem of antibiotic resistance. Despite the urgent needs for new antibiotics, only few companies are active in this field and a very limited number of compounds are under development [1] due to assumed low profits. This dilemma of antibiotic research demands for new strategies for efficient lower cost production on one side and possibilities to engineer existing antibiotics for new activities.

In order to narrow the gap between small molecule drugs (which encounter the problem of low target specificity) and larger biologics (which have limited oral bioavailability), peptide drugs are increasingly attracting wide interests as pharmaceutical agents [2]. Natural peptide products from nonribosomal and ribosomal synthetic sources have special structural and physicochemical properties which make them more accessible to pharmaceutical and agricultural implementations [3]. The plethora of information discerned from exploring uncultured microbes, as well as screening diverse resources and targets, suggest that bacteriocins could plausibly be applied as alternatives to conventional antibiotics based on their remarkable potencies against clinical targets [4]. There are clear evidences which suggest that class I bacteriocins (the lanthipeptides) have huge potentials in the health sector for future use as therapeutic agents [5].

Lanthipeptides are ribosomally synthesized, polycyclic natural peptide products that are usually characterized by the presence of the thioether cross-linked amino acids *(2S, 6R)*-lanthionine (Lan) or *(2S, 3S, 6R)*-3-methyllanthionine (MeLan) [6], as well as by additional α,β-unsaturated amino acids, such as dehydrobutyrine and dehydroalanine [7]. The stereochemistry of Lan and MeLan residues appear to be diversified; e.g. in the case of enterococcal cytolysin [8] the MeLan residues in the peptides have a special *(2R, 3R, 6R)* configuration. The structures of lanthipeptides derive from a precursor peptide by posttranslational modifications (PTMs) that occur within the core peptide, accomplished by modifying enzymes of the lanthipeptide gene cluster (*lan*). An *N*-terminal leader peptide is responsible for targeting the unmodified precursor to the modifying enzymes, and may also function as a translocation signal, as well as keeping the modified prepeptide inactive [9]. Due to their peculiar biosynthesis mode and their antibacterial activities, this group of bioactive peptides was first referred to as lantibiotics [6], however, owing to their diverse functionalities in targeting different categories of biological effects, they are presently referred to as lanthipeptides [10]. The polycyclic nature of lanthipeptides gives them an edge over other peptide compounds with respect to resistance to protease degradation [11] and limited conformational freedom which confers a high degree of target specificity [12].

**Fig. 1:**
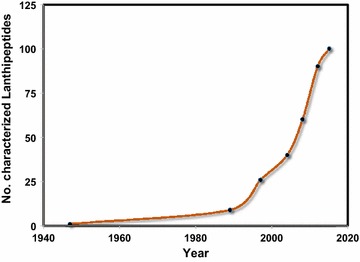
Growing number of characterized lanthipeptides over the years. The *graph* also presents data extracted from [15–17–18]

The number of identified lanthipeptides have increased tremendously during the last two decades; from 26 in 1997 [13] to more than 90 in 2012 [10], and to more than 100 characterized peptides in 2015 [14]. Figure 1 illustrates the number of isolated peptides over the years with the highest record between 2004 and 2012. While the interest in applying lanthipeptides as pharmaceutical agents is rising, there is a strong need in the development of the general platform methods for their production in the required amounts in order to meet the demands. Regrettably, the isolation of these natural products from their native sources is an expensive time-consuming process which results in very low yields in most cases.

Industrial peptide production is commonly based on three alternative technologies including solid-phase synthesis, liquid-phase synthesis, and in vivo biotechnological recombinant technology. Due to the complex nature of lanthipeptide structures, chemical synthetic strategies are challenging in economic terms, which leaves a huge space for the efforts of bioengineering in terms of industrial scale viability and generation of derivatives from the natural molecules in view of their clinical actions [19]. Moreover, applying bioengineering techniques on a heterologous host provides a tool also for the characterization of the biosynthetic routes, innovative ways of generating variants of the native product and increasing product yields where classical optimization of native strains has failed. Latest lanthipeptides reviews specializing on their antimicrobial potentials [5], mode of action [15], self-defensive strategies by producers [20], structure–activity relationships [21], and the importance of biosynthetic routes on discovery and bioengineering of novel peptides [10] have discussed the previous research in detail. We provide here a synoptic comparison of research efforts on total synthesis and biological procedures aimed at fostering the development of lanthipeptides production, which to our opinion have not been discussed anywhere in the literature. We further examine ongoing applications and analyze our present perception of current loopholes and propose measures to enhance production of these bioactive molecules using consistent bioprocess development strategies. Meanwhile chemical synthesis is a logical approach for the synthesis of these useful products, the scale-up process for large-scale production remains seemingly challenging due to difficulties in generating stereochemically accurate Lan and MeLan rings [22], not to mention the exceedingly low yields obtained in lab scales (Table 1). It is therefore highly likely that engineering robust biological systems for large scale production of these peptides is a particularly promising venture.

**Table 1:** Chemically synthesized lanthipeptides, analogues and mimics, with corresponding yields

Lanthipeptide, analogue or mimic (lab-scale synthesis)	Amount obtained (mg)	Product yield (%)	No. of steps	Approx. average yield per step (%)	References
Nisin	ND	0.003^a^	ND	70	[56]
Lactocin S	1.5	10	71	97	[57]
Lacticin 3147	4.8	1.0–1.4	>50	>95	[22]
Lacticin 481	2.0	1.3	52	92	[35]
Epilancin 15X	2.0	1.6	59	93	[58]
DAP-substituted ring A of lactocin S	1.5	2.5	>71	93	[44]
Norleucine substituted lactocin S	0.8	0.8	>70	ND	[59]
Nle, DAP-substituted lactocin S	1.0	1.8	>70	ND	[59]
N-terminal oxazole lactocin S	0.33	0.3	>70	ND	[59]
Alkene substituted lacticin 3147 Ltnβ	1.8	0.5	53	76	[40]
Oxygen substituted lacticin 3147	1.1	0.3	53	90	[41]
*Bis(desmethyl)*lacticin 3147	1.0	1.3	>50	84	[43]
LL- diastereomers of lacticin 481	1.9	1.3	>53	92	[35]
N-truncated variant of Epilancin 15X	1.2	1.9	45	92	[58]

*ND* data not described

^a^crude product yield before HPLC purification, obtained by calculating the overall yield of the five individually synthesized ring fragments and then the yield of putting these fragments together as performed by Shiba [56]

### Structural/functional properties that influence production of lanthipeptides

**Fig. 2:**
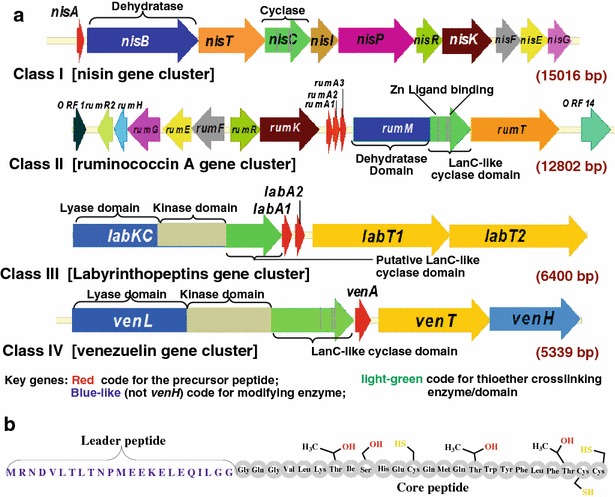
Summary of the major structural features in lanthipeptides biosynthesis and functions. **a** Schematic representation of one example of each class of lanthipeptide biosynthetic gene clusters, showing conserved motifs between the classes (indicated by *vertical lines* on designated genes with the *same colour*). Putative and characterized domains are respectively labeled: Class I (LanB, LanC), II (LanM), III (LanKC) and IV (LanL) processing enzymes install PTMs on their respective precursor peptides (LanA, cloured *red*). **b** Structure of ruminococcin A (LanA) precursor peptide showing the core peptide attached to the leader peptide and the side chains of amino acid residues that are targeted by RumM for lanthionine ring formation

Lanthipeptides were first classified by Jung based on structural characteristics into A and B types [23]. However, the discovery of novel complexities in those isolated thereafter prompted Sahl’s reclassification based on the structural similarities of the biosynthetic machinery [24]. The scheme proposed by Pag & Sahl was further developed by Willey and van der Donk in 2007 to include homology of the leader sequence, structure of the biosynthetic cluster, as well as the activity of the mature peptide [25]. Depicted in Fig. 2a, lanthipeptides can be classified into four distinct groups namely: class I where a dehydratase (LanB) and a cyclase (LanC) are involved in the formation of the PTMs; class II modified by LanM; class III modified by LanKC; and class IV by LanL [10]. The ‘Lan’ is a generic notation which symbolizes proteins that are encoded by the *lan* gene cluster and are involved in the biosynthesis and translocation of lanthipeptides. Unlike LanM, LanKC lacks the residues that are believed to coordinate the Zn^2+^ ion in the catalytic core of the lanthionine cyclase, suggesting an alternative catalytic pathway which should be further investigated. The latter information as well as the reversibility of lanthionine cyclase catalysis [26] could be useful for growth media engineering and possible thermodynamic control of the cyclisation reactions which may further improve active production of lanthipeptides in selected process schemes.

The biosynthetic machinery of lanthipeptides predominantly includes a two-component regulatory system with the histidine kinase LanK and the regulatory protein LanR. The ABC (ATP-binding cassette) transporter/processing protein LanT coordinates the modifying enzymes with the synthesis of the channel and thus is important for the translocation of the bioactive peptides from the cell to the extracellular environment where they perform their intended functions. The leader peptide, which in most cases is attached to the core peptide (Fig. 2b), is subsequently removed, after the core peptide has been modified as mentioned earlier, by a dedicated serine protease LanP prior to secretion of the active molecule out of the cell [27]. In other examples like nisin [28] and epidermin [29], the leader peptide cleavage and hence activation is performed extracellularly. With the exception of cytolysin, lanthipeptides which possess the Gly–Gly cleavage site in their precursor peptide are processed by an *N*-terminal protease domain of the LanT transporters [30]. Cytolysin undergoes a second cleavage/activation by a dedicated serine protease (CylP) [31] in the extracellular space after exportation by its processing protein (CylT). The LanFEGH proteins which are characteristic of the class I and class II lanthipeptides perform immunity roles by forming ABC-transporters that protect the cells from being attacked by the synthesized product [15, 32].

**Fig. 3:**
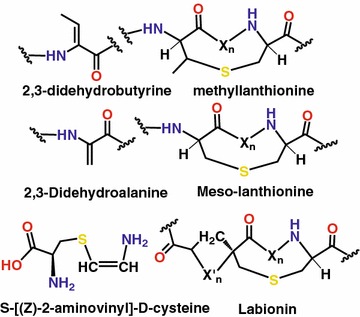
Major PTM features found in mature lanthipeptide. Didehydrobutyrine (Dhb) is derived from threonine and didehydroalanine (Dha) from serine. Thioether cross-linking between Dhb and cysteine results in methyllanthionine while that between Dha and cysteine gives lanthionine. The abbreviations X_n_ and X_n_’ represent peptide sequences

The PTMs labelled in Fig. 3 are distinctively unique, highly specialized in their biological functions [15], and amongst other factors such as self-immunity, make scale-up production of lanthipeptides in a heterologous host difficult since external hosts do not possess the essential requirements. Understanding the biosynthetic machinery of lanthipeptides has resolved diverse structural issues that have been useful in engineering lathionine/nonlanthionine-containing peptide variants (see below) as well as developing in vitro biosynthetic techniques [33]. The latter approach could moreover, facilitate production of compounds with very complex structures that are not readily amenable to the large-scale chemical synthetic process.

## Chemical synthesis

Previous and actual studies on lanthipeptide production have focused on the total chemical synthesis since their relatively small sizes (commonly <40 amino acids) is attainable with this technique. Moreover, the physicochemical properties of the generated product can be controlled via chemical synthesis by incorporating novel structural moieties [34] that could significantly enhance the pharmaceutical applicability. Additionally, the chemical synthesis is independent of the complex biological pathways [35] but instead relies on a standard technology. Thus chemical methods have been widely used to explore structure–activity relationships and to decipher the mode of action of various lanthipeptides. Over the last decade, information derived from these studies resulted in impressive achievements in developing this class of compounds, with a number of compounds which are currently under investigation or have completed phase I or phase II clinical trials, such as mutacin 1140, microbisporicin, actagardine and duramycin.

### Chemically synthesized derivatives; their activities and potential uses

Chemical synthesis and semisynthetic refinements of parent compounds are amongst the most popular tools used to generate the vast pool of conventional antibiotics [36], and could convincingly be well exploited to produce small compounds like vancomycin and clindamycin which also pose some structural challenges that limit practical large-scale synthetic approaches [37].

**Fig. 4:**
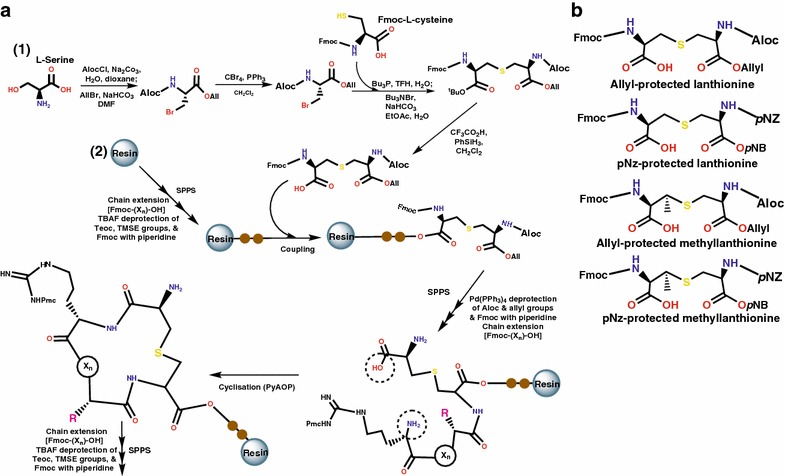
Simplified scheme, exemplifying chemical synthesis of lanthionine ring using OPLs. **a** General synthetic scheme of lanthionine ring of lanthipeptide involves dehydration of allyl protected serine or threonine and subsequent Michael addition reaction that couples an allyl protected cysteine to the dehydrated residues to form an OPL or orthogonally protected methyllanthionine respectively (1) The resultant OPL is further coupled to the growing peptide chain on a resin and processed via SPPS to incorporate the desired sequence (X_n_). Finally, cyclization reaction involving the encircled groups completes the ring formation, followed by SPPS and so on (2) The Allyl (All) protecting groups, *Fmoc* fluorenylmethyloxycarbonyl, *Pmc* penta-methylchromane, *Aloc* allyloxycarbonyl are essential in the process. The letter *R* (designated side chain). **b** Chemical structures of OPLs currently exploited for solid support synthesis of lanthipeptides

The first orthogonally protected β-methyllanthionine was synthesized by VanNieuwenhze in 2005 [38], followed by the solid-phase synthesis and use of orthogonally protected lanthionine (Fig. 4a_1_) to create a nisin ring C analogue by Tabor’s group [39]. These achievements opened exciting opportunities for solid-phase peptide synthesis (SPPS) of lanthipeptides (Fig. 4a_2_), since it was now possible to create orthogonally protected variants of lanthionine and methyllanthionine (Fig. 4b) with the right stereochemistries. Vederas’ group pioneered the synthesis of the non-lanthionine-containing carbocyclic analogue of Lacticin 3147 β-peptide using SPPS [40]. Lacticin 3147 is a two-component lanthipeptide consisting of two post-translationally modified components α and β, which act in synergy to accomplish their biological function, but their individual activities are limited. The Lacticin 3147 β-peptide variant however, did not show any activity even in synergy with the complementary α-peptide. Nevertheless, replacing the sulfur atoms of the tricyclic β-peptide of the two-component lanthipeptide with oxygen atoms [41] resulted in a weak biological activity. These strategies may be of wider use to enhance the pharmacokinetic properties of other peptides in this category in view of e.g. intestinal absorption, oral bioavailability, distribution and half-life. Such substitutions may also reduce the susceptibility of the compound to oxidation as shown in some cases [42]; much further investigation is required to evaluate the interactions of such analogues with human cells. The vulnerability of the lanthionine rings to oxidation may cause some technical constraints on therapeutic application of lanthipeptides which however, could be resolved by rationally exploring other derivatives.

**Fig. 5:**
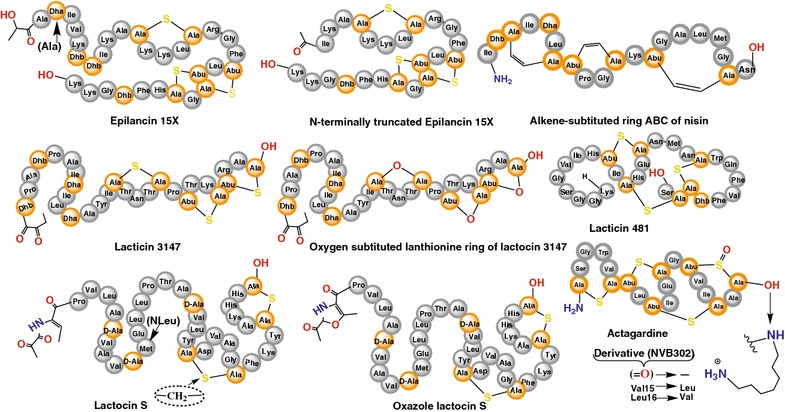
Illustrative examples of chemically synthesized analogues of lanthipeptides. Posttranslationally modified residues in natural synthesis hosts systems are highlighted. *Abu* aminobutyrine

The synthesis of *bis(desmethyl)* lacticin 3147 A2, realized by replacing the MeLan rings with Lan ring structures [43], also was a remarkable achievement with regards to synergism. Contrary to the alkene and oxygen substituted β-peptide, this variant displayed antimicrobial activity only in the presence of the complementary α-peptide. Here, the chemical synthesis provided an important tool to structurally differentiate between the separate inherent actions of the α- and β-peptides, respectively, and their synergistic activity. Similarly, a diaminopimelate (DAP)-substituted analogue of lactocin S (Fig. 5) was highly stable while retaining its full biological activity [44]. Recent investigations also succeded in coupling lipid moieties to the *C*-terminal A/B rings of nisin to produce semisynthetic constructs with potent activities against methicillin-resistant *Staphylococcus aureus* (MRSA) and vancomycin-resistant enterococci (VRE) [45]. All these analogues however, require thorough characterization for their safe application in humans, animals and food.

Tabor has discussed the various methods that have been developed to produce derivatives of orthogonally protected lanthionines and mythyllanthionines in her recent review papers [46, 47]. The techniques developed by Vederas group allow the synthesis of stereochemically accurate lanthionines and methyllanthionines [22]. These strategies are easily amenable to solid supported syntheses and could be further improved in large-scale scenarios to facilitate automation of the lanthipeptide synthetic process. Kirichenko and coworkers investigated the schemes created by Liu and colleagues to develop a technique that enables the synthesis of a series of overlapping intramolecular lanthionine bridges in polypeptides [48]. The so-called differentially protected orthogonal lanthionine technology (DPOLT) has been applied by Oragenics Inc. to develop a synthetic version of mutacin 1140, also called MU1140-S, which entered the preclinical phase to combat MRSA, *Clostridium difficile*, *Mycobacterium tuberculosis*, VRE and *anthrax* about half a decade ago [49]. Oragenics Inc. in collaboration with Intrexon Corp. and Bachem Americas Inc. established a joined effort to achieve the commercial production of MU1140-S [50]; according to a recent report, preclinical investigations on a lead clinical candidate showed an outstanding antibiotic efficacy [51].

Interestingly, the semisynthetic derivative of actagardine, NVB302, which is more globular in nature and differs by two amino acids in comparison to native actagardine (Fig. 5), has been created [52]. NVB302 was modified to exclude the sulfoxide bond present in the parent molecule, and also a diaminoalkane was introduced at the C-terminus. These modifications make the variant more soluble and active compared to the parent compound. Novacta Biosystems Ltd. began the clinical development of NVB302 [53], which has currently completed phase I clinical trials for the treatment of infections caused by *C. difficile* [54]. The same company also has registered patent rights for diaminoalkanes formulation of this variant into appropriately coated capsules that allow their oral administration since the modified product ensures dosage integrity through the gut to the desired intestinal region [55]. It is evident that chemical synthesis can be used in structure–activity relationship studies as well as for the production of stable lanthipeptides and semisynthetic variants with enhanced medical significance.

### ‘Proof-of-concept’ approaches for the full length synthesis of lanthipeptides

SPPS is currently the key technology for the production of peptides and smaller proteins with compositions that are not accessible by in vivo technologies for research and even for pharmaceutical applications. This technique had a tremendous impact on the synthesis of lanthipeptides over the last decade though the challenges posed by structural complexities still prevail [46]. The first solution phase synthesis of lanthipeptides was realized by Shiba and colleagues for nisin [56]. Examples that involve SPPS include the full-length synthesis of lactocin S [57], the total syntheses of both peptides of lacticin 3147 [22], analogues of epilancin 15X [58], and very recently lacticin 481 and its analogues [35]. Examples of some of the chemically synthesized peptides and their respective analogues are shown in Fig. 5, and product yields are described in Table 1.

Regardless of the achievements in total synthesis, the stereoisomeric relevance of biologically synthesized products and limits of chemical methods to incorporate all PTMs found in lanthipeptides are major concerns with this procedure. Considerable efforts are still needed to disclose ways to tackle these issues. An example which illustrates this challenge has been published for the synthesis of lacticin 481 analogues where the biological function was lost when the DL-Lan/MeLan ring configurations were replaced with the LL-stereoisomers [35]. The results in this article show that the enzymatically installed lanthionines are essential for the peptides to retain full activity. PTMs such as the labionin rings of labyrinthopeptins and the *S*-aminovinyl-d-cysteine of the epidermin group are difficult to synthesize chemically. In fact, an attempt to synthesize the labionin rings proved to be challenging [60], and though chemical synthesis of the (*Z*)-Aminovinyl-d-Cysteine structure has been realized for Mersacidin [61], the method uses expensive materials such as palladium (Pd) catalysts which could consequently significantly increase the cost of end product.

### Economic feasibility of the chemical synthesis of lanthipeptides

Although industrial scale chemical synthesis has been realized for a number of peptide drugs since many years, the industrial scale synthesis of lanthipeptides seems not to be profitable yet [62], practically due to expensive raw materials and overall low yields which culminates into exorbitant production costs in large-scale scenarios. The total synthesis of nisin resulted in 0.003 % yield of crude product [56] and that of lactocin *S* gave an overall yield of 10 % of the pure product in 71 reaction steps [57]. Subsequent synthesis of epilancin 15X, lacticin 3147, lacticin 481 and their analogues all resulted in product yields in the range of 0.3–2.5 % as shown in Table 1. Though the average product yields for each chemical step are operationally persuasive (70–97 %), the number of steps (in average between 45 and 75) are laborious and time-consuming if the amounts of pure substances obtained through lab-scale synthesis (0.33–4.8 mg) are taken into account (Table 1).

An example for a peptide which is successfully produced by chemical synthesis is Enfuvirtide (Fuzeon, Hoffmann-La Roche) which is a potent anti-HIV (human immunodeficiency virus) drug. Its application is very expensive with a daily consumption of $56.42 for EU member states [63] and an annual cost estimate of about $25,000 per patient undergoing Enfuvirtide therapy in the United States. Enfuvirtide is however, a relatively small peptide (36 amino acids) with an *N*-terminal acetylation and a C-terminal amidation as the only modifications [64]. The SPPS process for Enfuvirtide production involves 44 reagents and 106 reaction steps with an overall yield of 30 % [65]. 1 kg of Enfuvirtide is obtained from 45 kg of raw materials [63]. The elaborate nature of the manufacturing process coupled with the complex structural properties of the peptide are the reasons for the very high prices on the market, which is one of the restrictions for the wider use of this drug. While Enfuvirtide is a relatively simple peptide, lanthipeptides like nisin for example, have seven PTMs (including 5 lanthionine rings, Dhb and Dha) that makes their synthesis far more complicated. The average product yields obtained for lanthipeptides on a lab-scale so far are too low, entailing a more expensive final product on a commercial scale compared to Fuzeon, and their application as antibiotics does not justify a high price. In summary, chemical synthesis could be a faster approach to tackle pharmaceutical production, but process scale-up is so far not economically feasible and hence, biological production seems to be the only realistic alternative. This is perhaps only true for natural occurring structures since no efforts have been made on scale-up synthesis ever since the anticipated collaboration of Oragenics with Bachem Americas to produce Mutacin 1140-S in large scale yielded rather unexpected results [66], since Bachem only succeeded in generating improved yield of components required to synthesize MU1140-S, but could not complete the process of generating the full compound due to unexpected need for additional research.

## Biochemical synthesis and in vivo bioengineering

A wide range of biologically engineered systems and optimization strategies are currently used for the production of lanthipeptides, but only few industrial implementation studies are described. The major advantages of the biological systems include comparably high product concentrations, generation of the compounds in their right conformational geometries and very few downstream processing steps. Increasing knowledge and understanding of the key roles of various components in the biosynthesis of lanthipeptide, as described earlier, have provided enough insights on how bioengineering can be employed to suitably modify native strains and heterologous organisms to develop robust systems with enhanced production capabilities. Previous research on such approaches has been discussed in recent reviews [14, 62]. The successes of these techniques in developing process scale production and current applications are discussed herein.

### Current and potential applications of bioengineered lanthipeptides

**Fig. 6:**
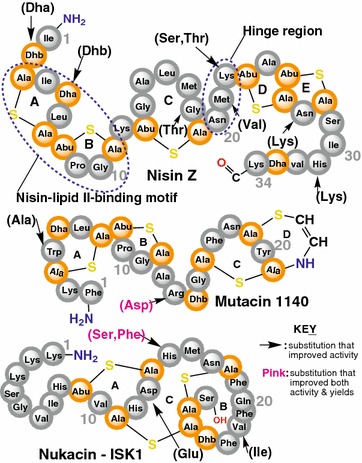
Illustrated examples of in vivo engineered lanthipeptides. The structures of nisin (showing the nisin-lipid II binding motif and the hinge region which are highly implicated in the activity of the compound), Mutacin 1140 and Nukacin-ISK1

The very first isolated lanthipeptide nisin has a high efficacy against multidrug-resistant pathogens on one side, and it showed a very low cytotoxicity and negligible incidence of resistances under routine applications on the other side. However, the therapeutic use of nisin was restricted by its low stability (intestinal protease degradation) and its reduced solubility at physiological pH [67]. This explains why improving the therapeutic relevance of lanthipeptides is equally important in the developmental process. Bioengineering of lanthipeptides is the major procedure currently used to modify their pharmacokinetic qualities and to improve their bioavailability as therapeutic agents. In the area of activity enhancement, one of the first variants of nisin *Z* obtained by replacing the Dhb at position 2 with Dha (Fig. 6) resulted in a two-fold increase of the activity compared to the native peptide [68]. Furthermore, the Lys22Thr/Ser derivative of nisin showed a higher bioactivity and reinforced specificity against *Listeria monocytogenes*, *S. aureus* and *Streptococcus agalactiae* [69], while the nisin A Met21Val mutant showed extensive activities against *C. difficile*, *L. monocytogenes,* MRSA and VRE [70].

Additionally, replacing the tripeptide Ile-Dha-Leu of the A ring of nisin with Val-Phe-Gly resulted in a derivative with enhanced activity against the native producer [71], which constitutes a possible disadvantage for in vivo production. Nevertheless, clinically relevant bacteria species often possess multidrug resistance ABC transporter complexes [72, 73], whose mechanisms of action are somewhat similar to the immunity ABC transporters found in the lanthipeptide producing organisms, and thus it is conceivable that this knowledge could be utilized to create peptides that would escape bacterial resistances. Similar mutagenesis studies and comparable results have also been obtained for nukacin-ISK1 [74], actagardine A [75] and lacticin 3147 [76]. Moreover, also a system for creating mersacidin derivatives with high potencies against MRSA, VRE and *Streptococcus pneumoniae* [77] was developed via in vivo engineering. Though the bioengineered lanthipeptides have positive effects in view of their bioactivity on the targets, little or no report has been described for scale-up production. Further investigations are definitely needed to develop strategies which enable efficient large-scale production with yields which are well in the gram per liter range as a basis for widespread pharmaceutical application.

Also the physicochemical properties of lanthipeptides have been dealt with accordingly. One good example is the nisin V Dha5Dhb variant that showed a high stability to chemical decomposition at low pH, as well as the Asn27Lys and His31Lys derivatives that both had a four–eight fold higher solubility compared to the natural compound at pH 7 [78]. These properties could allow the variants to be applied at high salts concentrations. All these results illustrate how in vivo engineering can be used to create peptides with improved pharmacokinetic properties.

Product yields of some lanthipeptides have also been enhanced via in vivo engineering. For example, the mutacin 1140 Arg13Asp variant [79] and the His15Ser mutant of nukacin-ISK1 [74] both showed improved product yields. Another report suggests that modifying the native producer to restrict the cleavage of the leader peptide has a significant positive influence on gallidermin yields and obviously decreases the toxicity of the product to the host [80]. This result was in corroboration with Jung’s suggestion in 1991 [81], subsequently verified by van der Meer and coworkers in 1994 [82] and very recently by Escano and colleagues [27], that the peptide is only active when the leader peptide has been cleaved off. These understandings can pave the way through a consistent bioprocess development for lanthipeptide production since several parameters can be evaluated on the basis of product sufficiency with respect to host strain’s integrity.

Meanwhile the leader peptide is an essential element for lanthipeptide bioengineering, although in downstream processing it constitutes some disadvantages since it has to be removed from the core peptide. Accordingly, some researchers have dedicated their efforts to engineer leader peptide independent modifying enzymes, such as the case of LctM, which resulted in an improved analogue of lacticin 481 [83]. Another recently described strategy is based on using amber stop codon suppression technique to incorporate hydroxy acids between the leader peptide and the core peptide, which introduces an ester bonds that can be simply and specifically hydrolyzed by alkaline hydrolysis after the peptide has been heterologously expressed in *Escherichia coli* [84]. This report is very important since it offers a very simple solution for the removal of the leader peptide from the modified precursor. Moreover, other methods for removing the leader peptide involves mutating the residue at −1 position of the propeptide to Arg, Lys, or Glu, which allows the use commercial proteases such as trypsin, Lys-C, or GluC [9, 83, 85–88]. Nevertheless, this method has some disadvantages; e.g. mutations can alter the morphology of the precursor peptide resulting in low expression levels in heterologous hosts, decreased processing of the precursor by the modifying enzyme, and unspecific removal of the leader peptide after the core peptide is modified. Additionally, the presence of such commercial protease sites in the core peptide would definitely limit the application of this procedure.

### Combined bioprocess strategies and bioengineering strategies for increased product yields

Lanthipeptides like many other bacteriocins are mostly produced by Gram-positive bacteria that are frequently found in many commercially useful products [89]. Nature has evolved in a way so that these toxins are produced in minute quantities by the native host, primarily to be used as a defensive target against competition in their ecological community. Therefore, direct cultivation and isolation of the biomolecules from their natural sources is challenging. Moreover, some native producers require very expensive medium components for growth, but studies have shown that such a situation can be prevented by engineering cultivation media; e.g. in the case of gallidermin the medium costs could be lowered by 93 % [90] and the yields were impressive as well. Whereas optimization of mutacin 1140 in minimal medium resulted in a hundred fold increase in yield compared to standard cultivation procedures [91], the outcome of this study only indicated that much efforts are required if large-scale amounts of the product are required (Table 2). Generally, the use of complex growth media to produce bacteriocins is the way for large-scale production of peptides which are used in the food industry for economic reasons [92], but due to variations in the ingredients it is challenging to fulfill the requirements for pharmaceutical production with complex media. Therefore, exploring alternative media formulations is an interesting area to focus research on. Curiously, a mixed culture system involving grain-based extracts was used to increase nisin production [93] and later, a simulation-based study projected yield of about 20 g L^−1^ fermentation broth using sweet whey and yeast extract as feedstocks [94]. These studies indicate that lanthipeptides could be successfully produced by simply using abundant and inexpensive raw materials.

**Table 2:** Selected lanthipeptides, production method and current concentration range recorded for each designated method

Lanthipeptide	Attainable [product] (mg L^−1^)					
	Native producer (mg/l)	Ref.	Optimized native producer (mg/l)	Ref.	External host (mg/l)	Ref.
Nisin	0.178	[95]^a^	1.09	[95]^a^	24	[96]
Lichenicidins	ND		ND		6	[19]
Prochlorosins	5.0 × 10^−4^	[96]	ND		1–3.5	[96]
Epilancin 15X	0.5	[97]	3.0	[98]	ND	
Epidermin	20	[99]	1000	[99]	ND	
Gallidermin	5.0	[100]	720, *200*	[90, 101]	ND	
Nukacin ISK-1	1.06	[102]	ND		1.5	[103]
Ala(0)actagardine	6.85	[104]	ND		4.2	[105]
Mutacin 1140	0.1	[91]	50, *10*	[91, 106]	ND	
Lacticin 481	0.6	[107]	1.1	[108]	ND	
Lacticin 3147	1.0	[109]	ND		ND	

Values were obtained from previous experiments, with special emphasis on their highest recorded yield. Entries in *italic* indicate yields obtained from engineering growth media to reduce cost

*ND* data not described

^a^value converted to mg L^−1^ using International Unit Converter (http://www.etoolsage.com/converter/IU_Converter.asp)

Optimizing physicochemical parameters such as pH and oxygen [106], changing magnetic fields in bioreactor [110], and using a packed-bed bioreactor to immobilize native producer organisms [111] all were strategies to increase product yields in natural hosts. Furthermore, an online recovery method that utilized silicic acid to adsorb nisin from cultivation medium also resulted in great improvements in yield [112]. Moreover, an optimized system was developed where the entire biosynthesis pathway of nisin was cloned into a plasmid, and expressing this plasmid in a closely related nisin deficient heterologous host showed a sixfold increase in yield (Table 2) compared to the native producer strain [95]. This kind of system could be very useful in producing those variants which tend to be toxic to producers [71]. Although optimizing production in some native producers has resulted in increased yields for some lanthipeptides under large-scale conditions, others have been unsuccessful as discussed in the next paragraphs.

### Economic viability of bioproduction processes for lanthipeptides

Economically, the amounts of lanthipeptides obtained from the cultivation of the natural producers are rather too low to be viably commercialized as pharmaceutical products. For instance, the cultivations of *Lactococcus lactis* yielded nisin in the range of 16-20 mg L^−1^ [16] under standard growth conditions. Similar outcomes were observed for gallidermin [100], epidermin [99], Lacticin 481 [107], Lacticin 3147 [109], nukacin ISK-1 [102], epilancin 15X [97], prochlorosins [96], Ala(0)actagardine [104] and mutacin 1140 [91], where the product yields ranged between 5.0 × 10^−4^ and 20 mg L^−1^ of culture. Although further optimization of the native strain consolidated approximately a thousand folds increases in yields for epidermin [99], gallidermin [101] and nisin [113], others were unsuccessful such as in the case of mutacin 1140 [106], epilancin 15X and lacticin 481 [98], where the product yields still remained in the range obtained by standard methods as presented in Table 2.

While all the reports described above focused on native producer strains, only a few studies have been performed on heterologous hosts probably due to restricted access to genetic information which are however readily available in the post genomic era. It is fascinating that engineered production in *E. coli* significantly improved yields compared to those obtained from the natural source of nisin [96]. Also other lanthipeptides were successfully produced in *E.coli* such as prochlorosins [114] and, very recently, lichenicidin [19]. Nonetheless, the production levels of nukacin ISK-1 [103] and Ala(0)actagardine [105] in *E. coli* were lower than those observed in their respective natural hosts (see Table 2). However, with *E. coli* as the production host, it is obvious that scale-up studies are economically feasible since no elaborate and sophisticated experimental settings are required.

The process for producing microbisporicin (NAI-107) by its native producer, *Microbispora corallina*, is the first reported case for industrial production of a lanthipeptide for use in humans as therapeutic drug. In this process an optimized cultivation medium is used at a 250 litre scale [115] to obtain kilogram-scale titers [116]. Food grade nisin (containing 5 % pure nisin) is currently produced by Zhejiang Silver-Elephant Bio-Engineering Co. at a production capacity of 100 metric ton per year and sold at US $100 per kilogram. The example of nisin is an indication that the development of stable biological production processes of pharma grade lanthipeptides is possible and only needs more research efforts. All the other compounds presented in Table 3 are research-grade qualities that are only produced in small quantities by the companies listed; and the prices are too high for common applications. In order to meet the requirements of a novel therapeutic agent, a production process for pharma grade lanthipeptides should accumulate costs that warrant a price competition between the final products and commercially available drugs.

**Table 3:** Selected lanthipeptides produced via biochemical means and current marketed prices by respective companies

Lanthipeptide (% purity)	Company	Price [US $ (×10^6^)/g]
Ruminococcin-A (>90 %)	MyBioSource	1.8
Lacticin-481 (>90 %)	MyBioSource	1.8
Mutacin 1140 (>98 %)	NovoPro	0.2486
Epidermin (>98 %)	NovoPro	0.2486
Nukacin ISK-1 (>98 %)	NovoPro	0.3051
Plantaricin W (>98 %)	NovoPro	0.3273
Lacticin 3147 (>98 %)	NovoPro	0.339
Bovicin HJ50 (>98 %)	NovoPro	0.3729
Actagardine (>98 %)	Santa Cruz Biotechnol	0.315

## Pharmaceutical prospects

Biologically produced nisin has been used as a food preservative for more than five decades without any significant incidence of microbial resistances [10]. Clinical studies have demonstrated possible uses of nisin as antimicrobial films on implantable medical devices [117], treatment for bovine clinical mastitis caused by *S. aureus* [118, 119] and mastitis in humans [120]. Nisin–ceftriaxone and nisin–cefotaxime have also been investigated for prospective use as adjunct therapies in the treatment of infections caused by *Salmonella* [121, 122]. Nisin can also significantly reduce biofilm formation of *Enterococcus faecalis* in combination with penicillin, ciprofloxacin, and chloramphenicol [123]. Very recently, nisin has been shown to reduce cell proliferation in head and neck squamous cell carcinoma (HNSCC) as well as in vivo tumorigenesis and hence could potentially be used as a novel therapeutic for HNSCC [124]. Additionally, high potency of a nisin V has been shown in connection to various chemotherapies [125]. Moreover, nisin V in combination with colistin, has been shown to reduce cytotoxic effects of the very potent polymyxin antibiotic to mammalian cells [126]. In veterinary medicine, nisin is applied in the therapeutic treatment of bovine mastitis [118] and is currently produced by ImmuCell Corporation (Maine, USA) under the brand name Wipe Out^®^ for the treatment of mastitis of lactating cows.

Duramycin from cultured cells has been produced and marketed by Durvet (Kansas, USA) for quite a while as an antibiotic for livestock. *In vitro* studies on nasal epithelium demonstrated that duramycin increases the transport of chloride ions [127], fluid secretions [128], and enhances the permeability of nasal membrane to chloride ions in healthy individuals and patients with cystic fibrosis [129]. Duramycin therefore has been proposed for the treatment of viral infections, cancers, dry eye syndrome and cystic fibrosis [130]. In 2007, a report on a phase II clinical studies on aerosolized duramycin (Moli1901) for the treatment of cystic fibrosis revealed exciting results [131]. This drug, which is jointly developed by Lantibio (North Carolina, USA) and AOP Orphan Pharmaceuticals (Vienna, Austria) [132] has completed phase II clinical trials for the treatment of cystic fibrosis [133].

The desire to improve the marketability issues in order to facilitate commercialization of lanthipeptides is quite encouraging as observed by the activities of some pharmaceutical companies. Just recently, Oragenics (Florida, USA) announced a successful pre-investigational new drug, based on their newly bioengineered lantibiotic called OG253 [134]. Novacta Biosystems Ltd. on the other hand secured the IP for the bioengineered system for creating mersacidin derivatives with robust activities against multidrug resistant microorganisms [77]. Meanwhile, microbisporicin is presently under clinical development by New Anti-Infective Consortium Scrl (Milan, Italy) and Sentinella Pharmaceuticals (New Jersey, USA) [135] for the treatment of *C. difficile,* MRSA and VRE [136]. At the moment, no lanthipeptide is produced and commercialized for therapeutic usage in humans [116] and therefore developing production processes that can sustainably deliver the products at high quality and prices that are comparable to conventional antibiotics is still interesting.

## Consistent bioprocess development strategies

A recent publication has described a novel technique where multiple genes from a lanthipeptide biosynthesis cluster were isolated and reconstructed in a simple surrogate expression system, *E. coli* [19]. This system allows recombinant production and secretion of the biomolecules out of the cell into the cultivation medium. Thus, developing scale-up schemes which seek to achieve overproduction of target bioactive compounds at industrial scale processing could be really interesting. Rational high through-put bioprocess optimization using mini fed-batch cultivation strategies could be employed to enhance stable productivity of such strains. Fed-batch technologies exist, that allow controlled *E. coli* cultivation conditions already in 96-microwell plates, ensuring high cell densities and greater yield of target products [137, 138]. Such techniques conceivably present an attractive area that could be explored to develop robust scalable bioprocess systems for producing lanthipeptides. This is possible since scaling up a suitably optimized micro scale cultivation will require less effort. A good example showing the strength of this method, has been recently reported for the non-ribosomally synthesized peptide, valinomycin. The biosynthetic pathway of the natural product was reconstituted in *E. coli* and optimized to obtain a yield of more than 10 mg L^−1^ in lab scale experiments [139] and greater than 2 mg L^−1^ under large scale processing [140]. Report elsewhere also indicate that environmental factors such as pH and aeration may have severe impact on the activities and product yields of lanthipeptides [141]. These heterogeneous scenarios could also be rigorously investigated in a timely and cost-effective manner, by applying e.g. the EnBase^®^ cultivation system [138] together with high through-put techniques such as cultivation in 96-Well sensor plates or mini-bioreactor systems [142] in automated liquid handling platforms.

## Conclusions

The lack of stable, high-yield, well-characterized processes and generally applicable procedures for sustainable production limit the availability of lanthipeptides for clinical studies and further pharmaceutical commercialization. In order to successfully develop and apply therapeutic lanthipeptides, the ultimate need for mass production processes to commercialize the product is very important. Ideally, chemical synthesis would produce a variety of structural analogues and also serves as a substitute method for producing large quantities of the desired product in cases where biological methods have been unsuccessful. However, the structural complexity of lanthipeptides and the combination of multiple complex synthetic steps make chemical production of the biomolecules a challenging and painful endeavor. This further complicates the establishment of stable chemical processes for large-scale synthesis.

Moreover, producing the structural elements of lanthipeptides in their stereochemically relevant configuration (which could facilitate the automation of the synthetic schemes) are not obviously trivial. In a nutshell, the elaborate structure of lanthipeptides, the exorbitant costs of chemical reagents, and the resultant overall low yields make chemical synthetic processes for lanthipeptides not economically viable at the moment. Additionally, a chemical synthetic process is not environmentally friendly due to toxic substances produced such as dichloromethane, dimethylformamide and trifluoroacetic acid during operation; such situations could be averted by using biological production procedures.

Comparatively, lanthipeptides like nisin and duramycin are currently produced through biological processes for use in food and livestock, respectively. Lab-scale production optimization of some lanthipeptides shows attractive improvements for some native producers. Some variants created through in vivo mutagenesis remarkably enhanced production and moreover, a 250 L scale industrial production of microbisporicin as a therapeutic agent against infections caused by MRSA has been successful. These successes indicate the usefulness of bioprocessing in targeting the manufacture of commercially viable therapeutic lanthipeptides. However, a general inability to achieve a production scale which allows for sufficient quantities of pure amounts of the molecules via standard lab-scale cultivation is not delightful. The results in terms of yields are somewhat disappointing. In order to overcome the challenges associated with product sufficiency and commercialization of lanthipeptides, cell engineering would be the most productive alternative. In this direction, heterologous hosts such as *E. coli* could be a relatively cheaper and faster option to develop a robust production process for large-scale applications. Such engineered systems should be developed in a way to ensure expression of the desired product on one side while minimizing the metabolic load on the host on the other side.

High throughput bioprocess screening and optimization of such engineered strains would ultimately lead to improved yields with limited labour costs. Complex impurities that could interfere with downstream processing may be avoided by the use of defined media and by designing strains that allow the target product to be secreted and purified from the culture supernatant. Moreover, once the optimization steps are done, scale up for mass production is much easier and requires less time and effort. Peptide natural products from strictly anaerobic sources like *Ruminococcus gnavus* which are difficult to cultivate require a surrogate host expression system for the establishment of a production process. The necessity for high-throughput techniques to effectively screen the isolated products cannot be underestimated.

Biological production and chemical syntheses both have useful assets that can be utilized via semisynthetic approaches to generate complex structural formulations in the bioactive peptides with enhanced therapeutic and pharmacological properties. However, process development is necessary to achieve industrial scale amounts, which based on available data is only feasible through biological procedures.

## Abbreviations

ABC: ATP-binding cassette
Abu: aminobutyrine
Dha: didehydroalanine
Dhb: didehydrobutyrine
DPOLT: differentially protected orthogonal lanthionine technology
HNSCC: head and neck squamous cell carcinoma
Lan: lanthionine
*lan*: genes involved in lanthipeptide biosynthesis
MeLan: methyllanthionine
MRSA: methicillin resistant *Staphylococcus aureus*
Nle: norleucine
OPL: orthogonally protected lanthionines
PTM: posttranslational modifications
SCBT: Santa Cruz Biotechnology^®^, Inc
SPPS: solid-phase peptide synthesis
VRE: vancomycin resistant Enterococci
